# A general quantum algorithm for numerical integration

**DOI:** 10.1038/s41598-024-61010-9

**Published:** 2024-05-07

**Authors:** Guoqiang Shu, Zheng Shan, Jinchen Xu, Jie Zhao, Shuya Wang

**Affiliations:** Laboratory for Advanced Computing and Intelligence Engineering, Zhengzhou, 450001 China

**Keywords:** Quantum algorithm, Numerical integration, Phase estimation, Quadratic acceleration, Physics, Computational science

## Abstract

Quantum algorithms have shown their superiority in many application fields. However, a general quantum algorithm for numerical integration, an indispensable tool for processing sophisticated science and engineering issues, is still missing. Here, we first proposed a quantum integration algorithm suitable for any continuous functions that can be approximated by polynomials. More impressively, the algorithm achieves quantum encoding of any integrable functions through polynomial approximation, then constructs a quantum oracle to mark the number of points in the integration area and finally converts the statistical results into the phase angle in the amplitude of the superposition state. The quantum algorithm introduced in this work exhibits quadratic acceleration over the classical integration algorithms by reducing computational complexity from *O*(*N*) to *O*(√*N*). Our work addresses the crucial impediments for improving the generality of quantum integration algorithm, which provides a meaningful guidance for expanding the superiority of quantum computing.

## Introduction

Numerical integration is a classical and important problem with a wide range of applications in many fields of physics, chemistry, biology, computer and finance^1–3^. However, existing classical integration algorithms face high computational complexity, O(*n*), where *n* represents the number of points used for integration calculation over the interval. Fortunately, quantum algorithms exhibit acceleration superiority over classical algorithms in some problems. For example, Shor’s integer factorization algorithm, which shows exponential acceleration over classical algorithms^4^. Grover’s algorithm has quadratic acceleration over the classical algorithms in solving unordered database search problems^5^. The HHL algorithm for solving systems of linear equations also exhibits exponential acceleration and is widely used in machine learning and other fields^6^. Nevertheless, a general quantum algorithm for numerical integrations is still absent.

Definite integrations are commomly approximated by classical numerical methods. For example, interpolation integration formulas include Newton–Cotes formula, Complex quadrature formula, Romberg formula and Gauss formula^7–9^. The algebraic accuracy of Newton–Cotes formula and Complex quadrature formula is *n*, where *n* is the number of interpolation nodes. But when *n* > 7, these methods lose their effectiveness. The algebraic accuracy of Romberg formula is 2*n*, and the algebraic accuracy of Gauss formula is 2*n* + 1. These interpolation integration formulas utilize the *n*-order polynomials value at *n* interpolation points to approximate the integrations, and their complexity is O(*n*). Besides that, the monte carlo integration (MCI) method generates *n* random numbers in the integration area S. The number of random numbers in the integration area is *m*, thus the integration is approximately equal to *m*S/*n*. The complexity of MCI method is O(*n*) and error is *O*(1/√*n*)^10^, which the error can be reduced by key sampling and Quasi-monte carlo method, etc.^11,12^. R. P. Kanwal et al. presented an integration method using taylor expansion with the precision *n*^13,14^ and complexity O(*n*). In all, the complexity of these classical algorithms is O(*n*) that is high compared with quantum algorithms.

For taking advantage of quantum acceleration to reduce the complexity of classical algorithms, some quantum algorithms were peoposed. For example, Acioli et al. proposed a Quantum Monte Carlo (QMC)^15,16^ integration algorithm with quadratic acceleration for periodic functions^17^. Abrams et al. proposed a fast quantum integration algorithm by using the grover's mean and quantum counting, which obtains exponential speed acceleration and quadratic acceleration in comparison with classical deterministic and probabilistic methods respectively, however, the function is required to be discrete Boolean type in this method^18^.

Shimada et al. presented the quantum coin algorithm based on the quantum supersampling algorithm achieving quadratic acceleration^19,20^, but the value of the functions is limited to [0,1]. Heinrich raised a quantum integration algorithm with quadratic acceleration for Sobolev-like high-dimensional function^21^. DeWitt-Morette et al. proved a quantum algorithm with exponential acceleration over deterministic classical algorithms on the functions of Holder class^22^. Heinrich proposed a quantum integration algorithm on the Lebesgue space, which proves the algorithm is optimal^23^. Rebentrost et al. presented an optical quantum multi-dimensional integration algorithm, which demonstrates quadratic acceleration over MC method^24^. These algorithms mentioned above have limitations on the type of integration function. In addition, some quantum and classical hybrid integration algorithms have also been proposed, such as Suzuki et al. raised a hybird integration method to simplify the circuit depth^25–27^. Moreover, some are controversial over acceleration capability, such as the MCI method based on quantum amplitude estimation (QAE) can bring quadratic acceleration^28,29^, but Kaneko et al. confirmed that the probability distribution of the initial state coding prepared by the Grover-Rudolph method does not reflect the quantum advantage^30^.

Existing classical integration algorithms face high computational complexity and quantum algorithms face low generality problems, as shown in Table 1. Here, we propose a general quantum integration algorithm (GQIA) that eliminates these shortages showing strong universality and operability. Firstly, our algorithm quantizes the classical Monte Carlo integration process and utilizes quantum superposition to possess exponential representation capabilities beyond classical methods. Secondly, to construct a quantum oracle with polynomial approximation of the integration function, use the parallelism of quantum to count the points on the integration region, and store them in the phase of the quantum state. Finally, amplitude amplification and phase estimation are used to obtain phase information with high probability and accuracy, whereupon the integration value is calculated. Compared with classical integration algorithms, GQIA demonstrates the quadratic acceleration and higher computational accuracy. Due to the use of Monte Carlo and polynomial approximations, GQIA has no limitations on the formal properties of integration functions, making it more versatile than other quantum algorithms.

**Table 1:** Performance analysis of different integration algorithms.

Quantum algorithm			
	Suitable area	Complexity	Precision
GQIA	Any continuous function	$O\left((1/\epsilon)\sqrt{N/M}\right)$	$\mathcal{O}{\left(\frac{\sqrt{N}-1}{\sqrt{N}\left({x}^{l+1}\right)\epsilon }\right)}^{1}$
Algorithm-1^31^	Holder function	$O\left({\left({\text{log}}{\varepsilon }^{-1}\right)}^{1/(1+\gamma )}\right)$	$\mathcal{O}{\left({n}^{-\alpha /(d-1)}\right)}^{2}$
Algorithm-2^21^	Sobolev function	$O(n)$	$\mathcal{O}{\left(1/{n}^{-r/(d-1)}\right)}^{3}$
Algorithm-3^23^	Lebesgue function	$O\left( {(1/\varepsilon )^{{\frac{p}{{2\left( {p - 1} \right)}}}} } \right),$ $1 \le p < 2$; $O\left( {\left( {1/\varepsilon } \right)} \right),2 \le p \le \infty$	$\mathcal{O}\left({n}^{-2+2/p}\right)$
Algorithm-4^18^	Any continuous function	$O(n)$	$\mathcal{O}\left({n}^{-1}\right)$
Photonic quantum^24^	Multi-dimensional integrations	$\mathcal{O}\left((1/ \varepsilon) \right)$	$\mathcal{O}{\left(1/{\epsilon }^{\delta }\right)}^{4}$
Quantum monte carlo^17^	Periodic function	$O\left( {n^{2 - \delta } } \right),0 \le \delta \le 1$	$\mathcal{O}(1/\sqrt{n})$
Quantum coin metho^20^	Function takes the value [0,1]	$O(n)$	$\mathcal{O}(1/\sqrt{n})$
Quantum supersampling^19^	Boolean Function	$O(n)$	$\mathcal{O}(1/n)$

Classical Algorithm			
	Suitable Area	Complexity	Precision
Gauss forum^7–9^	High precision with fewer nodes	$O(n)$	$\mathcal{O}\left(1/{x}^{2n+1}\right)$
Taylor expansion^13,14^	Each derivative of the function is known	$O(n)$	$\mathcal{O}\left(1/{x}^{n}\right)$
Romberg forum^7–9^	less computation, high precision requirements	$O\left({n}^{k}\right)$	$\mathcal{O}\left(1/{x}^{2k}\right)$
Monte carlo^10^	Complex function or the form is unknown	$O(n)$	$\mathcal{O}\left({1}/{\sqrt{n}}\right)$
Composite rule^7–9^	Degree $<8$ and integration interval is large	$O(n)$	$\mathcal{O}\left(1/{x}^{n}\right)$
Newton–cotes forum^7–9^	Degree $<8$	$O(n)$	$\mathcal{O}\left(1/{x}^{n}\right)$

^1^*N* stands for the number of points of area S, *N* = 2^k1^^+^^k1^ , and has the same meaning as other *n* in the table. *M* represents the number of point in the area S_1_.

^2^$\alpha$, $d$ is real number.

^3^$r$, $d$ is real number.

^4^$\delta$ is a constant that can be arbitrarily small.

## Methods

For more comprehensive discussions of GQIA, we begin here by briefly reviewing some relevant results from quantum algorithm and quantum computation theory.

### Amplitude amplification

The key idea of the amplitude amplification (AA) algorithm^24^ originally came from an unordered database-search algorithm, known as Grover’s quantum algorithm^5^. By amplifying the amplitude of a given pure state, the algorithm achieves the purpose of adjusting the measurement probability of the pure state. The specific implementation method of the algorithm is given as follows, first given an oracle ${\hat{\mathcal{A}}}$ and initial state $|0{\rangle }^{\otimes n}$

1 $$\left| {\left. \psi \right\rangle =\hat{\mathcal{A}} |0{\rangle }^{\otimes n}={\hat{\mathcal{A}}}} \right|\left. {00 \ldots 0} \right\rangle = \cos \theta /2\left| {\psi_{0} \rangle} \right. + \sin \theta /2\left| {\psi_{1} \rangle} \right.$$

where $\theta \in [0,\pi ],\left|{\psi }_{1}\right.\rangle$ is the target pure state and $\left|{\psi }_{0}\right.\rangle$ is the non-target pure state. The state vector $|\psi \rangle$ can be expressed as a parameterized vector $({\text{cos}}\theta /2,{\text{sin}}\theta /2)$ on the space stretched by two basis vectors $\left|{\psi }_{0}\right.\rangle$ and $\left|{\psi }_{1}\right.\rangle$. The purpose is to promote the amplitude of the $\left|{\psi }_{1}\right.\rangle$ first, while reducing the amplitude of the non-target state, because the sum of the squares of the two probability amplitudes is 1. At this time, the measurement probability of the target state will also increase. This is done by flipping the quantum state $|\psi \rangle$ towards the direction of the target state $\left|{\psi }_{1}\right.\rangle$. i.e., making $\theta$ as much larger as possible.

In the AA algorithm, one amplitude amplification requires two flips. The first flip uses the unitary operation ${\mathbf{S}}_{\chi }$ to flip the state $|\psi \rangle$ along $\left|{\psi }_{0}\right.\rangle$ and change state $|\psi \rangle$ to $-|\psi \rangle$. The second flip uses the unitary operation $2|\psi \rangle \langle \psi |-I$ to flip the first result ${\mathbf{S}}_{\chi }|\psi \rangle$ along $|\psi \rangle$. The amplitude of the final target state $\left|{\psi }_{1}\right.\rangle$ becomes larger in the resulting state $(2|\psi \rangle \langle \psi |-I){\mathbf{S}}_{\chi }|\psi \rangle$.

The overall operation $\hat{Q}$ is defined as

$$\begin{gathered} \left| {\left. {\psi_{{\text{result }}} } \right\rangle } \right. = \hat{Q}\left| {\left. \psi \right\rangle = \left( {2\left| {\left. \psi \right\rangle \left\langle \psi \right.} \right| - I} \right)S_{\chi } } \right|\left. \psi \right\rangle \hfill \\ \quad \quad \quad \; = \left( {2{\hat{\mathcal{A}}}\left| {\left. {00 \ldots 0} \right\rangle \left\langle {00 \ldots 0} \right.} \right|{\hat{\mathcal{A}}}^{ - 1} - I} \right)S_{\chi } |\left. \psi \right\rangle \hfill \\ \quad \quad \quad \; = {\hat{\mathcal{A}}}\left( {2\left| {\left. {00 \ldots 0} \right\rangle \left\langle {00 \ldots 0} \right.} \right| - I} \right){\hat{\mathcal{A}}}^{ - 1} S_{\chi } |\left. \psi \right\rangle \hfill \\ \end{gathered}$$

where $(2|00\dots 0\rangle \langle 00\dots 0|-I)$ operation means to flipping the amplitude of all quantum states but keeping the amplitude of $|00\dots 0\rangle$ unchanged. By repeating this process many times, the quantum state $|\psi \rangle$ can be flipped to the target state $\left|{\psi }_{1}\right.\rangle$ with an angle of $\theta$ each time. What we need to pay attention to is that the real value of $\theta$ does not need to be known in advance.

### Phase estimation

Quantum phase estimation is an algorithm for estimating the phase information of quantum states, and it is the core of many quantum algorithms. Two quantum registers are necessary in the phase estimation circuit, which the first register requires $t$ qubits whose initial state is $|0\rangle$ and the value of $t$ depends on two aspects: the number of digits for precision and successful probability of phase estimation result.

The initial state of the second register is the prepared state $|u\rangle$ and the number of qubits is as many as possible to store $|u\rangle$. The process of phase estimation is roughly divided into three steps. First, the circuit begins with $t$ Hadamard gates that are applied to all qubits in the first register, and the controlled- $U$ operations are applied simultaneously on the second register, where the $U$-gate appears in consecutive integer powers of 2. The resulting state is

2 $$\frac{\left(|0\rangle +{e}^{2\pi i{2}^{t-1}\varphi }|1\rangle \right)\left(|0\rangle +{e}^{2\pi i{2}^{t-2}\varphi }|1\rangle \right)\dots \left(|0\rangle +{e}^{2\pi i{2}^{0}\varphi }|1\rangle \right)}{{2}^{t/2}}=\frac{1}{{2}^{t/2}}\sum_{k=0}^{{2}^{t}-1} {e}^{2\pi i\varphi k}|k\rangle$$

The second step of phase estimation is the application of inverse quantum Fourier transform on the first register, and this step can be accomplished in $O\left({t}^{2}\right)$ stages. The third and final step of phase estimation is to measure the state in the first register and get an estimation of $\varphi$. The fianl state of the first register can be written as

3 $$\frac{1}{{2}^{t/2}}\sum_{j=0}^{{2}^{t}-1} {e}^{2\pi i\varphi j}|j\rangle |u\rangle \to |\tilde{\varphi }\rangle |u\rangle$$

where $\tilde{\varphi }$ denotes the estimator for $\varphi$ when measured.

## Proposed methods

### A General quantum integration algorithm (GQIA)

**Figure 1:**
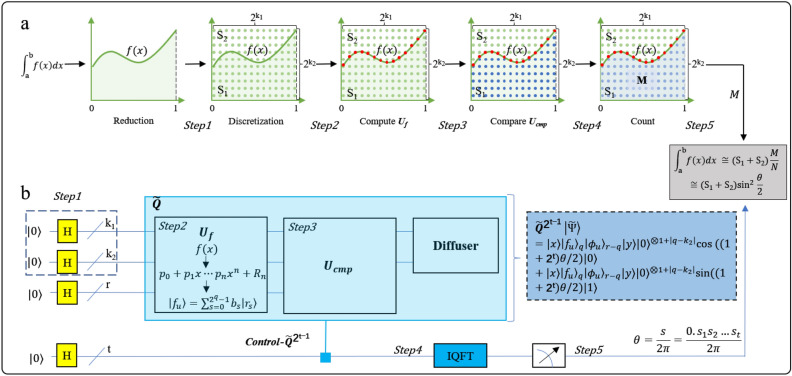
Diagram of quantum integration algorithm GQIA. (**a**) The classical monte carlo integration(MCI), where *S*_1_ represents integration area, *S*_2_ stands for non-integration area. *M* stands for the number of points in the area *S*_1_, and *N* is the total points in *S*_1_ + *S*_2_. (**b**) Quantum circuit diagram of GQIA, *Step*1–*Step*5 corresponds to quantization of the MCI.

We define *S* as the area consisting of integration area *S*_1_ and non-integration area *S*_2_. *N* and *M* denote the number of points in *S* and *S*_1_ respectively (Fig. 1a). The core idea of GQIA is to convert a definite integration problem on a continuous interval [*a, b*] into the problem of getting the value of N*${{\text{sin}}}^{2}(\theta /2)$ with quantum computing. The phase *θ* that contained in the amplitude of a superposition state is obtained by phase estimation, as shown in Fig. 1b. To achieve integration calculation with quantum method, we need to address the following challenges.

The first problem is to encode and calculate integrable functions with quantum gate circuits. Supposing the function *f* (*x*) is integrable in the interval [*a, b*], it can be written as ${\int }_{a}^{b}f(x)=F\left(b\right)-F\left(a\right)=I$ and *I* is a known constant. However, when functions are complex or cannot be formulated, they are usually approximated by the linear combination of function values under some discrete points in the interval.

4 $${\int }_{a}^{b} f(x)dx\approx {\sum }_{k=0}^{n} {A}_{k}f\left({x}_{k}\right)$$

The integration function can be quantized by quantum coding under discreate variables after selecting appropriate approximation methods.

The second problem is to get the distribution of points in the area *S*_1_. The point *x* that meets *f* (*x*) ≤ *y* is required, and the number is recorded as *M*. By constructing quantum gate circuits of computation and comparison, the distribution of these points in *S*_1_ is acquired and stored in the amplitude of a superposition state.

The third problem is to get the ratio λ of distribution. There is no acceleration advantage to measure result from quantum circuit directly. Thus, by phase estimation circuit, we can acquire the phase *θ* with quadratic quantum advantage and the approximate value of the integration is S*${{\text{sin}}}^{2}(\theta /2)$.

### The components of GQIA

#### Quantization of integration functions

Quantization of integration functions refers to the quantum representation of classical integration functions and the construction of the quantum circuits. The integrations over any interval [*a*, *b*] can be transformed into interval [0, 1], and the real number points (*x*, *y*) in the area *S* can be discretized by using *k*_1_ and *k*_2_ qubits, respectively. This means using bits of length *k*_1_ to represent numbers between 0 and 1, similarly, to using bits of length *k*_2_ to represent the value of the vertical axis *y* and these points evenly divide the integration interval *S*.

5 $$x,y) = \left\{ {\left( {x_{i} ,y_{ij} } \right)|i = {1},{2}, \ldots ,{2}^{{k_{1} }} ; j = {1},{2}, \ldots ,{2}^{{k_{2} }} } \right\}$$

Thus, the number of discreate points is *N* = 2^k1^^+^^k1^ (Fig. 1a).

The researches on quantum integration algorithms are rare, and one of the obstacles is to represent integrable functions with quantum coding. In numerical analysis, continuous and bounded functions can be polynomials approximated, such as Chebyshev approximation, best square approximation^8^, etc. If the function *f*(*x*) has *n*-order derivatives on interval [*a*, *b*] including *x*_0_, function *f*(*x*) can be approximated with a Taylor expansion at point *x*_0_, which the coefficients are the *n*-order derivatives of the function at *x*_0_. Otherwise, the polynomial coefficients can be determined by taking the function values from multiple points and using the method of undetermined coefficients, and the function is approximated by a simple polynomial, as shown in Eq. (6)

6 $$f\left( x \right) = a_{0} + a_{{1}} * x + a_{{2}} * x^{{2}} + \cdots + a_{n} * x^{n} + R_{n} \left( x \right)$$

**Figure 2:**
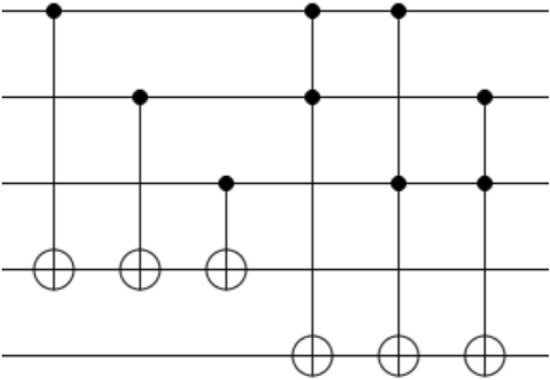
Quantum circuit of quadratic operation.

If the first *n* + 1 terms are used to approximate the function, the precision of approximation is *O*(1*/x*^*n*+1^). Polynomial approximation is a linear combination of the power of variables and when the value of *k*_1_ of variable discretization is determined, the power circuit is easy to construct, that is the reason for approximating integration functions with Eq. (6). For example, the quadratic power quantum circuit (Fig. 2) can be constructed with Table 2.

**Table 2:** Binary multiplication table.

*0*	*0*	*0*	*x*_3_	*x*_2_	*x*_1_
*0*	*0*	*0*	*x*_3_	*x*_2_	*x*_1_
*0*	*0*	*0*	*x*_1_ ∗ *x*_3_	*x*_1_ ∗ *x*_2_	*x*_1_
*0*	*0*	*x*_2_*x_3_	*x*_2_	*x*_1_ ∗ *x*_2_	*0*
*0*	*x*_3_	*x*_2_*x_3_	*x*_1_ ∗ *x*_3_	*0*	*0*
*0*	*c*_4_	*0*	*0*	*0*	*0*
*c*_5_	*c*_3_	*c*_2_	*c*_1_	*0*	*0*
*c*_5_	*c*_3_ + *c*_4_ + *x*_3_	*c*_2_	*c*_1_ + *x*_2_	*0*	*x*_1_

#### Construction of marking oracle

The area *S* is divided into *S*_1_ and *S*_2_ by the function curve, and only the points in *S*_1_ are required. Here, a method is proposed to mark these points by constructing two quantum oracles. The first one is *U*_*f*_ computing *f*(*x*), which $f \left(x\right)=\sum_{i=0}^{n}{a}_{i}{x}^{i}$ is the first *n* terms of the approximate polynomial.

Supposing *r* denotes the number of auxiliary qubits for this oracle, including *q* qubits representing the superposition output of *f* (*x*) under the initial input ${\left|{f}_{u}\right.\rangle }$. The oracle needs *k*_1_ + *r* qubits in that way, where *k*_1_ stands for the number of qubits for variable *x*, and $\left|{\phi }_{u}\right.\rangle$ is the output of the auxiliary qubits.

7 $$H^{{ \otimes k_{1} }} |0\rangle ^{{ \otimes k_{1} }} |0\rangle ^{{ \otimes r}} \mathop \to \limits^{{U_{f} }} \left( {\frac{1}{{\sqrt 2 }}} \right)^{{k_{1} }} \sum\limits_{{u = 0}}^{{2k_{1} - 1}} | u\rangle \left| {f_{u} } \right.\rangle _{q} \left| {\phi _{u} } \right.\rangle _{{r - q}}$$

**Figure 3:**
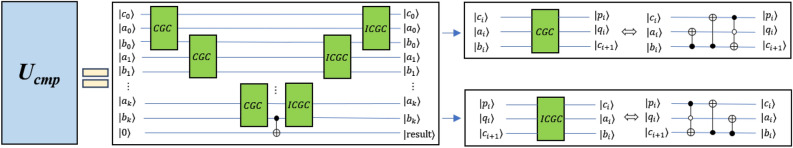
Structure and detailed circuits of comparator U_cmp_.

The other oracle is to compare *f*(*x*_*i*_) with *y*_*ij*_ for each *x*_*i*_. The point (*x*_*i*_, *y*_*ij*_) that meets *f* (*x*_*i*_) ≤ *y*_*ij*_ needs to be marked, and a comparison circuit including *n* CGC gates and *n* ICGC gates can make it^32^, which CGC and ICGC gates are quantum circuits composed of CNOT and Toffoli gates, and the CGC gate and its inverse ICGC are constructed with two CNOT gates and one Toffoli gate in different orders respectively (Fig. 3). Thus, the number of qubits is 2*n* + 2.

8 $$\begin{gathered} U_{{cmp}} U_{f} H^{{ \otimes k_{1} + k_{2} }} |0\rangle ^{{ \otimes k_{1} + k_{2} }} |0\rangle ^{{ \otimes r + 2 + \left| {q - k_{2} } \right|}} \hfill \\ \quad = U_{{cmp}} U_{f} |x\rangle |y\rangle |0\rangle ^{{ \otimes r + 2 + \left| {q - k_{2} } \right|}} \hfill \\ \quad = U_{{cmp}} |x\rangle \left| {f_{u} } \right.\rangle _{q} \left| {\phi _{u} } \right.\rangle _{{r - q}} |y\rangle |0\rangle ^{{ \otimes 2 + \left| {q - k_{2} } \right|}} \hfill \\ \quad = |x\rangle \left| {f_{u} } \right.\rangle _{q} \left| {\phi _{u} } \right.\rangle _{{r - q}} |y\rangle |0\rangle ^{{ \otimes 1 + \left| {q - k_{2} } \right|}} \sqrt {1 - \lambda } |0\rangle \hfill \\ \quad \quad + |x\rangle \left| {f_{u} } \right.\rangle _{q} \left| {\phi _{u} } \right.\rangle _{{r - q}} |y\rangle |0\rangle ^{{ \otimes 1 + \left| {q - k_{2} } \right|}} \sqrt \lambda |1\rangle \hfill \\ \end{gathered}$$

where ${k}_{2}$ is the number of qubits required to represent $y,|y\rangle ={\left(\frac{1}{\sqrt{2}}\right)}^{{k}_{2}}{\sum }_{\nu =0}^{{{2}{k}_{2}}-1} |\nu \rangle$, similarly, $|x\rangle ={\left(\frac{1}{\sqrt{2}}\right)}^{{k}_{1}}{\sum }_{u=0}^{{{2}{k}_{1}}-1} |u\rangle$. If $q$ is not equal to ${k}_{2},2+\left|q-{k}_{2}\right|$ auxiliary qubits $|0{\rangle }^{\otimes 2+\left|q-{k}_{2}\right|}$ are needed for this comparator. $\lambda$ is a real number between 0 and 1 evaluated by measurement, which representing the proportion of points that meet the comparison criteria and corresponding to ampulitude of the auxiliary bits, when the output is 1. Thus, the comparison results are obtained by evaluating the value of $\lambda$, and the necessary qubit number of marking oracle is ${k}_{1}+{k}_{2}+r+2+\left|q-{k}_{2}\right|$.

To sum up, the marking oracle $\mathbf{U}$ of GQIA is made up with computation and comparation, which can be recorded as $\mathbf{U}={U}_{cmp}{U}_{f}$. Different from direct measurement, the GQIA obtains the results by phase estimation, which brings quadratic acceleration^33^.

#### Extraction of results

For a quantum state in amplitude amplification (AA) algorithm^29^,

9 $$|\Psi \rangle =\mathcal{A}|0{\rangle }_{n}|0\rangle =\sqrt{1-\lambda }{\left|{\psi }_{0}\right.\rangle }_{n}|0\rangle +\sqrt{\lambda }{\left|{\psi }_{1}\right.\rangle }_{n}|1\rangle$$

where ${\left|{\psi }_{1}\right.\rangle }_{n}$ is the required target state, ${\left|{\psi }_{0}\right.\rangle }_{n}$ is non-target state. Defining $\theta \in [0,\pi ]$, so that ${{\text{sin}}}^{2}\theta /2=\lambda$ and a unitary operator ${\text{Q}}$,

10 $${\text{Q}}=-\mathcal{A}{{\text{S}}}_{0}{\mathcal{A}}^{-1}{ \, {\text{S}}}_{\chi }$$

${\mathbf{S}}_{\chi }$ adds a negative phase before ${\left|{\psi }_{1}\right.\rangle }_{n}|1\rangle ,{\left|{\psi }_{0}\right.\rangle }_{n}|0\rangle$ keeps unchanged.

11 $$|\Psi \rangle =\mathcal{A}|0{\rangle }_{n}|0\rangle ={\text{cos}}\theta /2{\left|{\psi }_{0}\right.\rangle }_{n}|0\rangle +{\text{sin}}\theta /2{\left|{\psi }_{1}\right.\rangle }_{n}|1\rangle$$

Using ${\text{Q}}$ repeated *j* times for the quantum state $|\Psi \rangle$ gives

12 $${{\text{Q}}}^{j}|\Psi \rangle ={\text{cos}}((2j+1)\theta /2){\left|{\psi }_{0}\right.\rangle }_{n}|0\rangle +{\text{sin}}((2j+1)\theta /2){\left|{\psi }_{1}\right.\rangle }_{n}|1\rangle$$

Similarly, from Eq. (8) we can get a quantum state $|\widetilde{\Psi }\rangle$ and a unitary operator $\widetilde{\mathbf{Q}}$ as

13 $$\begin{gathered} |\tilde{\Psi }\rangle \;\; = {\mathbf{U}}H^{{ \otimes k_{1} + k_{2} }} |0\rangle ^{{ \otimes k_{1} + k_{2} }} |0\rangle ^{{ \otimes r + 2 + \left| {q - k_{2} } \right|}} \hfill \\ \quad \quad = |x\rangle \left| {f_{u} } \right.\rangle _{q} \left| {\phi _{u} } \right.\rangle _{{r - q}} |y\rangle |0\rangle ^{{ \otimes 1 + \left| {q - k_{2} } \right|\sqrt {1 - \lambda } |0\rangle }} \hfill \\ \quad \quad \quad \; + |x\rangle \left| {f_{u} } \right.\rangle _{q} \left| {\phi _{u} } \right.\rangle _{{r - q}} |y\rangle |0\rangle ^{{ \otimes 1 + \left| {q - k_{2} } \right|\sqrt \lambda |1\rangle }} \hfill \\ \quad \quad = |a\rangle |0\rangle + |b\rangle |1\rangle \hfill \\ \end{gathered}$$

14 $$\widetilde{\mathbf{Q}}=-\mathbf{U}H{\mathbf{S}}_{0}{H}^{-1}{\mathbf{U}}^{-1}{\mathbf{S}}_{\chi }$$

where

15 $$|a\rangle =|x\rangle {\left|{f}_{u}\right.\rangle }_{q}{\left|{\phi }_{u}\right.\rangle }_{r-q}|y\rangle |0{\rangle }^{\otimes 1+\left|q-{k}_{2}\right|}\sqrt{1-\lambda }=|x\rangle {\left|{f}_{u}\right.\rangle }_{q}{\left|{\phi }_{u}\right.\rangle }_{r-q}|y\rangle |0{\rangle }^{\otimes 1+\left|q-{k}_{2}\right|}{\text{cos}}(\theta /2)$$

16 $$|b\rangle =|x\rangle {\left|{f}_{u}\right.\rangle }_{q}{\left|{\phi }_{u}\right.\rangle }_{r-q}|y\rangle |0{\rangle }^{\otimes 1+\left|q-{k}_{2}\right|}\sqrt{\lambda }=|x\rangle {\left|{f}_{u}\right.\rangle }_{q}{\left|{\phi }_{u}\right.\rangle }_{r-q}|y\rangle |0{\rangle }^{\otimes 1+\left|q-{k}_{2}\right|}{\text{sin}}(\theta /2)$$

In the ${\text{AA}}$ algorithm, the unitary operator $\widetilde{\mathbf{Q}}$ is equivalent to rotate the superposition states by angle $\theta$, which can be expressed as a 2*2 dimensional matrix in the single bit case

17 $$\widetilde{\mathbf{Q}}=\left[\begin{array}{cc}{\text{cos}}\theta & -{\text{sin}}\theta \\ {\text{sin}}\theta & {\text{cos}}\theta \end{array}\right]$$

It is easy to get the eigenvalues of $\widetilde{\mathbf{Q}}$ from following formula

18 $$|\gamma I-\widetilde{\mathbf{Q}}|=\left|\begin{array}{cc}\gamma -{\text{cos}}\theta & {\text{sin}}\theta \\ -{\text{sin}}\theta & \gamma -{\text{cos}}\theta \end{array}\right|=(\gamma -{\text{cos}}\theta {)}^{2}+{{\text{sin}}}^{2}\theta =0$$

The eigenvalues are ${\gamma }_{1}={e}^{i\theta }$ and ${\gamma }_{2}={e}^{i(2\pi -\theta )}$, either one is feasible because $\theta$ has a small value and $2\pi -\theta$ is large, making it easy to observe. In this article, it may be assumed that the value is $\theta$ and we take the case of ${\gamma }_{1}$. Phase estimation is to get $s$ in $\widetilde{\mathbf{Q}}|\psi \rangle ={e}^{2\pi is}|\psi \rangle$, where $s=\frac{\theta }{2\pi }$. Supposing that we denote $s$ with $t$ qubits, that is, $s=0.{s}_{1}{s}_{2}\cdots {s}_{t}$. We can obtain the result of with inverse quantum Fourier transform(Eq. 19).

19 $$\frac{1}{\sqrt{{2}^{t}}}\sum_{j=0}^{{2}^{t}-1} {e}^{2\pi isj}|j\rangle \to |s\rangle$$

Therefore, $s$ and $\lambda$ can be obtained by measuring the first $t$ qubits in phase estimation circuit, and the integration can be approximated with ${\lambda }^{2}\cdot N$.

20 $${\int }_{a}^{b} f(x)dx\approx \left({S}_{1}+{S}_{2}\right)\cdot {\lambda }^{2}={2}^{{k}_{1}+{k}_{2}}\cdot {{\text{sin}}}^{2}\left(\frac{\pi s}{{2}^{t}}\right)$$

## Experimental evaluations

To understand GQIA, we propose a simple study case ${\int }_{-1}^{1} {e}^{x}dx$.

First, the intrgration interval $[-\mathrm{1,1}]$ can be transformed to interval $[\mathrm{0,1}]$, and the result is

21 $${\int }_{-1}^{1} {e}^{x}dx=2{\int }_{0}^{1} {e}^{2z-1}dz$$

It is known that ${e}^{x}=1+x+\frac{{x}^{2}}{2!}+\cdots +\frac{{x}^{n}}{n!}+o\left({x}^{n}\right)$ with Taylor expansion, and ${e}^{x}$ can be approximated with the first three terms of Tylor polynomial. For special functions that cannot be Taylor expanded, other polynomial approximation methods could be available as mentioned in Section “Quantization of integration functions”.

22 $$2{\int }_{0}^{1} {e}^{2z-1}dz\approx {\int }_{0}^{1} 4{z}^{2}+1dz$$

**Figure 4:**
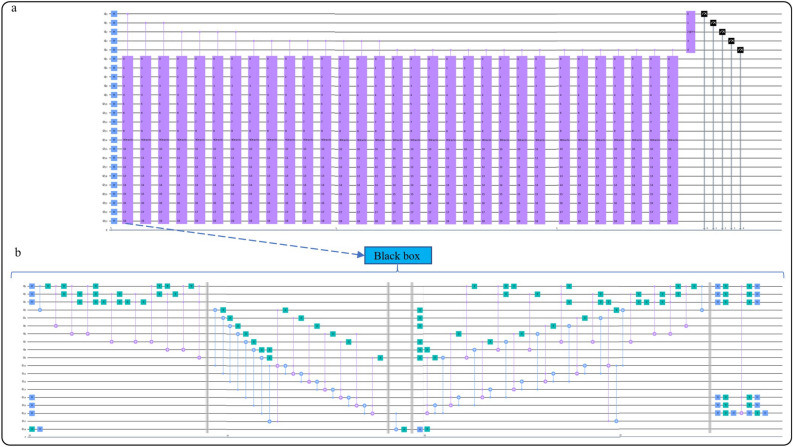
Diagram of ${\int }_{-1}^{1} {e}^{x}dx$ with GQIA. (**a**) The circuit of GQIA for the study case. (**b**) The black box(oracle) for the study case of GQIA.

Then, the oracle circuits that include calculation and comparation circuits are constructed to mark the points (Fig. 4b). We take the number of qubits for variable $z$ and $y,{k}_{1}=3$ and ${k}_{2}=3$ respectively, thus the number of qubits for function $4{z}^{2}+1$ is $q=7$ in Eq. (7), and the number of auxiliary qubit is $r=7$ in Eq. (8).

Finally, the phase $s=\frac{\theta }{2\pi }$ contains the result $M$ (Fig. 4a) is obtained by phase estimation and is estimated to $t=5$ qubits accuracy in Eq. (19). Hence, the total number of qubits is ${k}_{1}+{k}_{2}+r+t+2+\left|{k}_{2}-q\right|=24$, and the experiments were implemented with IBM qiskit 32-bit simulator qasm—simulator.

The final result is $\theta =4.52$, the number of the points in $S$ is $M=N\cdot {{\text{sin}}}^{2}\frac{\theta }{2}=$ ${2}^{6}\cdot {{\text{sin}}}^{2}\frac{\theta }{2}=25.76$, and the approximated value of integration is $6.125\cdot \frac{25.76}{{2}^{6}}=2.465$. In contrast, ${\int }_{-1}^{1} {e}^{x}dx$ = 2.333, thus the obtained precision is 0.943. According to the precision analysis in Section “Proposed methods”, ϵ ≤ 6.283, the experimental precision satisfies the lower bounds of precision $\frac{\sqrt{N}-1}{\sqrt{N}\left({x}^{l+1}\right)\epsilon }$ = 0.557. However, the precision of direct measurement fluctuates from 0.570 to 0.809 in the same situation, which demonstrates GQIA is more reliable and high-accuracy.

## Discussion

The whole process of GQIA is divided into three steps. The first step achieves quantization of any integrable functions by polynomial approximation and quantum encoding, the second step constructs the oracle of marking and the third step gets the results by the phase estimation.

The first step approximates integration function with the first $l+1$ terms of a simple polynomial. The number of qubits to compute the value of function $f(x)$ is ${k}_{1}+r$, where ${k}_{1}$ denotes the number of qubits for variable $x$, and $r$ is the number of auxiliary qubits including $q$ qubits to represent result $\left|{f}_{u}\right.\rangle$, where $q<r$. The computational precision is $\mathcal{O}\left(1/{x}^{l+1}\right)$.

In the second step, ${k}_{2}$ is the number of qubits for variable $y$. When ${k}_{2}$ is not equal to $q$, additional $\left|{k}_{2}-q\right|+2$ auxiliary qubits are required for the comparator circuit. Thus, the number of qubits in the first two steps is ${k}_{1}+{k}_{2}+r+\left|{k}_{2}-q\right|+2$, and the precision keeps unchanged, $\mathcal{O}\left(1/{x}^{l+1}\right)$.

In the third step, $t$ qubits are required to estimate $\theta$ to $m$ qubits accuracy, where $t=$ $m+\lceil {\text{log}}(2+1/(2\epsilon ))\rceil$. Thus, the number of required qubits is ${k}_{1}+{k}_{2}+r+\left|q-{k}_{2}\right|+2+t$. For getting the accurate value of $\theta$ with high probability $1-\epsilon ,\widetilde{{\text{Q}}}$ operator need to be implemented $\frac{\pi }{4}\sqrt{{2}^{{k}_{1}+{k}_{2}}/M}$ times, and the maximum execution number of $\widetilde{{\text{Q}}}$ does not exceed ${2}^{t-1}$ times. Hence, $t={\text{log}}\left(\frac{\pi }{4}\sqrt{{2}^{{k}_{1}+{k}_{2}}/M}\right)+c$, where $c$ is a constant related to error $\epsilon$ and the upper bound of error $\epsilon$ is $\frac{2\pi }{{2}^{t}}\sqrt{M(N-M)}+\frac{{\pi }^{2}}{{2}^{2t}}|N-2M|$. As a result, the complexity of GQIA is $O\left(\frac{1}{\epsilon }\sqrt{\left({2}^{{k}_{1}+{k}_{2}}/M\right)}\right)$ that is recorded as $O\left(\frac{1}{\epsilon }\sqrt{N/M}\right)$^33,34^, and the precision is $\mathcal{O}\left(\frac{\sqrt{N}-1}{\sqrt{N}\left({x}^{l+1}\right)\epsilon }\right)$.

## Conclusion

In conclusion, the GQIA we proposed for solving numerical integration, showing superiority of quantum algorithm in numerical problems. Quantum encoding of any integrable functions presents strong generality by approximating the functions with polynomials. Furthermore, constructing oracle and converting the results to phase exhibit the advantages of quadratic acceleration. The GQIA provides a quantum integration algorithm framework based on the MCI idea, where methods such as polynomial approximation, spatial discretization, and oracle construction are not unique and can be further optimized and studied.

The future work worth exploring about the algorithm of this article including the following points. The GQIA's circuit is relatively deep that poses challenges in running on current quantum computers, the depth of the circuit is mainly caused by phase estimation, and improvements or alternative algorithms can be studied. In addition, the polynomial approximation methods of the integration function may affect the complexity of GQIA's oracle circuits. For example, GQIA mainly applies the truth table method to construct circuits, and further research can be conducted on polynomial circuit construction methods.

## Data Availability

All relevant data supporting the main conclusions and figures of the document are available on request. Please refer to Guoqiang Shu at sstronger21@163.com.

## Code availability

The code used for obtaining the presented numerical results as well as for generating the plots is available from the corresponding author on reasonable request.
